# Sending Out an SOS: Mitochondria as a Signaling Hub

**DOI:** 10.3389/fcell.2016.00109

**Published:** 2016-10-13

**Authors:** Iryna Bohovych, Oleh Khalimonchuk

**Affiliations:** ^1^Department of Biochemistry, University of Nebraska-LincolnLincoln, NE, USA; ^2^Nebraska Redox Biology Center, University of Nebraska-LincolnLincoln, NE, USA; ^3^Fred and Pamela Buffett Cancer Center, University of Nebraska Medical CenterOmaha, NE, USA

**Keywords:** mitochondria, retrograde signaling, metabolites, reactive oxygen species, mitochondrial stress

## Abstract

Normal cellular physiology is critically dependent on numerous mitochondrial activities including energy conversion, cofactor and precursor metabolite synthesis, and regulation of ion and redox homeostasis. Advances in mitochondrial research during the last two decades provide solid evidence that these organelles are deeply integrated with the rest of the cell and multiple mechanisms are in place to monitor and communicate functional states of mitochondria. In many cases, however, the exact molecular nature of various mitochondria-to-cell communication pathways is only beginning to emerge. Here, we review various signals emitted by distressed or dysfunctional mitochondria and the stress-responsive pathways activated in response to these signals in order to restore mitochondrial function and promote cellular survival.

## Introduction

Mitochondria are semi-autonomous dynamic organelles of endosymbiotic origin involved in a plethora of vital functions including energy conversion and synthesis of key precursor metabolites, reducing equivalents, and cofactors. For more than half a century, mitochondria have been known to the general audience as the “powerhouse of the cell” (Siekvitz, 1957). This scientific meme refers to the organelle's most commonly known bioenergetic function—generation of adenosine triphosphate (ATP). This process commences by an uptake of substrates from the cytosol and is followed by their catabolic conversion via fatty acid oxidation and/or the citric acid (TCA) cycle, which yields reducing equivalents, nicotinamide adenine dinucleotide (NADH), and flavoadenine dinucleotide (FADH_2_) as well as multiple biosynthetic precursors. The reducing equivalents produced through the TCA cycle fuel the electron transport chain component of the mitochondrial oxidative phosphorylation system (OXPHOS), wherein flow of electrons through respiratory complexes is linked to generation of a proton gradient across the inner mitochondrial membrane (IM) that is required for ATP production by F_1_F_0_ ATP synthase. Although these functions are clearly crucial for cellular physiology and human health, the concept of mitochondria as isolated biosynthetic and bioenergetic units is insufficient to explain certain phenotypic outcomes or clinical manifestations associated with known mitochondrial dysfunctions.

The past two decades gave rise to tremendous research progress in the field of mitochondrial biology. They provided a gamut of evidence that mitochondria are deeply integrated into cellular physiology and metabolism. Mitochondria retain their own genome and transcription/translation machineries; however, because they co-evolved with their host cell, a number of originally mitochondrial genes have been transferred to the nucleus, thus contributing to the dual genetic origin of the mitochondrial proteome. That is, the vast majority of proteins comprising the mitochondrial proteome is encoded by nuclear DNA, synthesized in the cytosol, and is subsequently imported into the organelle. This bi-genomic nature of the mitochondrial proteome necessitates tightly coordinated expression of both mitochondrial and nuclear genes to produce stoichiometric amounts of its components to maintain proper organelle function. The signaling mechanisms that assure such communication are historically classified into anterograde and retrograde signaling. The former mechanisms mediate coordination of mitochondrial gene expression and—more broadly—mitochondrial function in response to endogenous and environmental homeostatic alterations sensed in the cytosol or by other organelles (usually the nucleus). In turn, retrograde signaling mechanisms monitor a variety of signals emitted by the mitochondria that allow for communicating the functional state (e.g., levels of energy production or the organelle's biosynthetic capacity) of mitochondria with other cellular compartments.

While much of the previous research focused on anterograde signaling, recent advances highlight and greatly expand the original paradigm postulated in the 1990's (Butow and Avadhani, 2004), whereby mitochondria are viewed as critical signaling hubs that take part in multiple cellular decisions. In this review, we will focus on molecular signals produced by the mitochondria to communicate homeostatic alterations and coordinate retrograde responses. We will survey several diverse groups of these signals including: (1) nucleotides; (2) precursor metabolites; (3) free radicals; (4) peptides and polypeptides; and (5) other molecules such as ions and lipids. Consequently, we will discuss their significance and impact on mitochondrial and cellular physiology.

## Signaling through mitochondria-derived nucleotides

While the generation of ATP is undoubtedly one of the major mitochondrial functions, mitochondria also provide some key precursors to other nucleotides or nucleotide-based reducing equivalents. It is therefore not surprising that cells have developed sensitive mechanisms to monitor the levels of these molecules and adjust cellular metabolic demands accordingly. In this section, we will discuss how alterations in mitochondrial or cellular levels of these mitochondria-borne molecules due to mitochondrial dysfunction or distress are recognized as an alarming signal to initiate a chain of events necessary to restore cellular energy and metabolic homeostasis.

### Adenylate nucleotides

**Figure 1:**
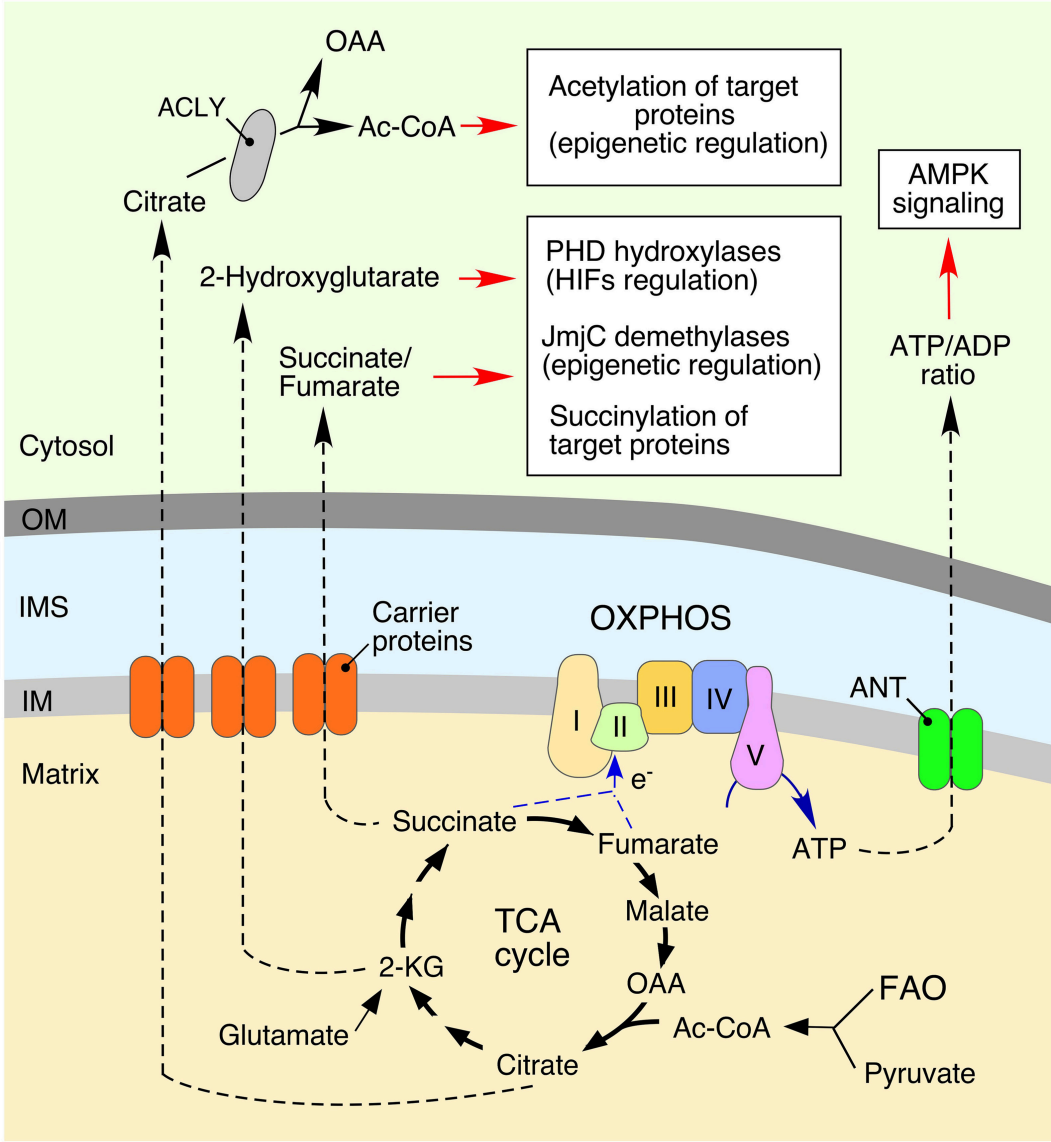
**Mitochondria-derived metabolites regulate various cellular processes**. Bioenergetic function of mitochondria is associated with generation of ATP (adenosine triphosphate) via the oxidative phosphorylation (OXPHOS) system. This process involves conversion of various substrates imported from the cytosol via the tricarboxylic acid (TCA) cycle or fatty acid oxidation (FAO). Metabolic intermediates can be exported into the cytosol to impact various signaling cascades. Mitochondria-derived citrate is converted to oxaloacetate (OAA) and acetyl coenzyme A (Ac-CoA) by ATP citrate lyase (ACLY). Subsequently, a fraction of the cytosolic Ac-CoA can be used in protein acetylation, thereby impacting multiple cellular processes (Choudhary et al., 2009; Spange et al., 2009; Wellen et al., 2009; Eisenberg et al., 2014). Another TCA cycle biosynthetic intermediate, 2-ketoglutarate (α-KG), can be converted to 2-Hydroxyglutarate. This metabolite inhibits: (1) Jumonji C domain-containing demethylases (JmjC), affecting epigenetic reprogramming (Pearce et al., 2013; Zong et al., 2016); and (2) hypoxia-inducible prolyl hydroxylases (PHDs), thus stabilizing the hypoxia-inducible factors (HIFs) and inducing a hypoxic response (Xu et al., 2011; Pearce et al., 2013; Zong et al., 2016). The TCA metabolite, fumarate, can also act as a signal-modulating factor via its ability to bind lysine residues of various proteins, resulting in a post-translational modification called succinylation. The adenine nucleotide translocator (ANT) mediates an exchange of ATP and cytosol-derived ADP (adenosine diphosphate) across the inner mitochondrial membrane (IM). Mitochondrial dysfunction derived ATP depletion perturbs ATP/ADP ratios by subsequent activation of adenosine monophosphate-activated protein kinase (AMPK), a master regulator of cellular energy homeostasis (Hall et al., 2013; Pearce et al., 2013).

Generation of ATP through mitochondrial oxidative phosphorylation is central to the maintenance of the optimal ATP/adenosine diphosphate (ADP) ratios within the cell (Figure 1). Under conditions of mitochondrial damage, decreased ATP production results in depletion of intracellular ATP, which leads to increased intracellular concentrations of adenosine monophosphate (AMP) or its subsequent derivative, adenosine. The latter nucleotide directly binds to the γ subunit of the energy-sensing adenosine monophosphate-activated protein kinase (AMPK) complex (Hardie et al., 2016). This regulatory binding acts in concert with an upstream regulatory protein kinase LKB1 to promote activation of AMPK (Pearce et al., 2013; Hardie et al., 2016). AMPK is a key metabolic sensor in the cell and its activation initiates multiple signaling events leading to a series of interconnected processes: (1) inhibition of ATP-dependent biosynthetic pathways to prevent wasteful use of cellular ATP; (2) stimulation of ATP production via catabolic reactions (Hall et al., 2013; Pearce et al., 2013); (3) activation of autophagic removal of damaged mitochondria (mitophagy) (Egan et al., 2011; Kim et al., 2011); and (4) metabolic re-tuning of mitochondria via promotion of mitochondrial fission (Toyama et al., 2016). These molecular events enable restoration of cellular energy homeostasis and allow cells to cope with metabolic distress.

### Nicotinamide adenine dinucleotide

Nicotinamide adenine dinucleotide is a vital redox molecule present in the cell in both oxidized (NAD^+^) and reduced (NADH) forms. One distinctive feature of NAD^+^ is that cells can utilize it as both a cofactor and a co-substrate (Canto et al., 2015; Verdin, 2015). Cells maintain several NAD^+^ pools in different cellular compartments; the mitochondrial NAD^+^ pool is segregated from the rest of the cell due to impermeability of the mitochondrial inner membrane. Just like in the case of adenine nucleotides, the NAD^+^/NADH equilibrium is central to normal cellular function. In mitochondria, the optimal NAD^+^/NADH and FADH/FADH_2_ ratios are primarily maintained through OXPHOS function and the TCA cycle. In addition, the cytosolic and mitochondrial NAD^+^ pools are connected via malate/aspartate and glyceraldehyde-3-phosphate shuttles that promote a mitochondria-cytosol exchange of reducing equivalents (Birsoy et al., 2015; Verdin, 2015). Perturbations of OXPHOS function decrease the NAD^+^/NADH ratio thereby creating a deficit in mitochondria, and, ultimately, leading to cytosolic NAD^+^/NADH imbalance (Birsoy et al., 2015; Sullivan et al., 2015). Because glycolytic enzymes are NAD^+^-dependent, respiratory compromised cells divert mitochondria-destined pyruvate toward lactate production—the reaction that also yields NAD^+^ for use in glycolysis. These actions can subsequently activate AMPK- and MAP kinase-mediated downstream signal transduction pathways (reviewed in Canto et al., 2015). Of note, elevated cellular levels of lactate usually reflect mitochondrial dysfunction and serve as a common diagnostic marker in patients with mitochondrial disease (DiMauro and Schon, 2003; Vafai and Mootha, 2012).

As mentioned above, NAD^+^ is also utilized as a co-substrate by several different classes of enzymes including sirtuin protein diacetylases—a group of versatile enzymes distributed across subcellular compartments and involved in regulation of various cellular activities such as histone modification and modulation of master transcriptional regulators like forkhead box 03 (FOXO3), nuclear factor kappa B (NF-κB), and peroxisome proliferator-activated receptor gamma co-activator 1 alpha (PGC-1α, Canto et al., 2015; Verdin, 2015). Alterations in cellular NAD^+^ levels may, therefore, lead to substantial changes in the activity of sirtuins and promote global transcriptional changes.

### Other nucleotides

Yet another nucleotide-based signaling mechanism is thought to include the mitochondrial inner membrane-anchored enzyme dihydroorotate dehydrogenase (DHODH), which oxidizes dehydroorotate to produce orotate—the key precursor in biosynthesis of pyrimidine nucleotides, uridine triphosphate, and cytidine triphosphate (Evans and Guy, 2004). DHODH activity is critically dependent on OXPHOS activity and the presence of reduced ubiquinone as a direct electron acceptor. As such, a dysfunctional OXPHOS stalls *de novo* synthesis of pyrimidines, which in turn leads to stabilization and nuclear accumulation of the tumor suppressor protein p53 and subsequent transcriptional responses (Khutorenko et al., 2010).

## Mitochondrial biosynthetic intermediates and signaling

The mitochondrion is a source of several key metabolic precursors utilized by cellular biosynthetic pathways. There is growing evidence that mitochondria-produced intermediary metabolites are not just mere biosynthetic building blocks, but also potent regulators of various cellular signaling cascades. In general, most, if not all, of the examples of mitochondrial nucleotide-mediated signaling surveyed above can be viewed from a metabolic sensing perspective. However, for the reader's convenience, we chose to discuss them separately. Here, we will focus on TCA cycle-borne biosynthetic intermediates that may exert signaling functions.

### Acetyl coenzyme A

Acetyl coenzyme A (Ac-CoA) is a central donor of two-carbon units utilized in multiple biosynthetic reactions in the mitochondrion. While Ac-CoA, *per se*, is unable to cross mitochondrial membranes, its condensation with the four-carbon TCA cycle intermediate oxaloacetate by citrate synthase produces citrate that can be either utilized in subsequent rounds of the TCA cycle or be readily exported out of the mitochondria (Figure 1). In the cytosol, citrate is converted back to oxaloacetate and Ac-CoA, which can be used in lipid biosynthesis and protein acetylation. The latter post-translational modification is mediated by lysine acetyltransferases and can widely impact multiple cellular processes including signaling (Choudhary et al., 2009; Spange et al., 2009; Wellen et al., 2009; Eisenberg et al., 2014).

Interestingly, Ac-CoA and citrate metabolism-related signaling have been in the spotlight for quite a long time. The first retrograde response mechanism identified in respiratory deficient yeast cells in 1990s invokes metabolic remodeling to replenish mitochondrial glutamate, citrate, and Ac-CoA pools (Liao and Butow, 1993; Liu and Butow, 1999). This apparently yeast-specific pathway, known as the retrograde response gene, or RTG pathway, includes a sensor protein, Rtg2, and two transcriptional factors, Rtg1 and Rtg3 that are kept inactive in the cytoplasm through phosphorylation. Mitochondrial dysfunction appears to be sensed by Rtg2, which promotes dephosphorylation and subsequent nuclear accumulation of the Rtg1/Rtg3 tandem, thereby triggering transcriptional responses. More details on the RTG pathway can be found in the following reviews (Butow and Avadhani, 2004; Haynes et al., 2013).

### 2-ketoglutarate, succinate, and fumarate

Succinate, fumarate and 2-ketoglutarate are the four-carbon and five-carbon metabolites of the TCA cycle, respectively. These molecules can be exported to the cytosol through dicarboxylate carrier proteins and utilized as the donors of carbon units in a variety of biosynthetic reactions (Figure 1). However, in certain mitochondrial dysfunctions—e.g., mutations affecting the function of the TCA cycle enzymes isocytrate dehydrogenase, succinate dehydrogenase, or fumarate hydratase—cells accumulate these metabolites in both the mitochondria and cytosol (Gaude and Frezza, 2014; Parker and Metallo, 2015; Zong et al., 2016). Accumulation of 2-ketoglutarate (2-KG) is associated with its conversion to 2-hydroxyglutarate (2-HG), a potent mimetic, and inhibitor of 2-KG-dependent dioxygenases in the cell—most notably hypoxia-inducible factor prolyl hydroxylases (PHDs) (Xu et al., 2011; Pearce et al., 2013; Zong et al., 2016) and the Jumonji-domain family histone lysine demethylases (Jmj-KDMs) (Pearce et al., 2013; Zong et al., 2016). Inhibition of these enzymes leads to significant epigenetic and transcriptional changes (Pearce et al., 2013; Zong et al., 2016). Additionally, 2-HG-mediated inhibition of PHDs stabilizes hypoxia-inducible factors (HIFs), thereby activating hypoxic signaling in the cell. It is therefore not surprising that 2-HG is a recognized oncometabolite and its accumulation has been reported in many cancers (Losman and Kaelin, 2013; Zong et al., 2016).

Accumulation of succinate appears to have a similar impact on function of PHDs and has been linked to tumorigenesis (Zong et al., 2016). Accumulation of fumarate results in the metabolite's binding to lysine residues of various proteins—so called succinylation (Zhang et al., 2011). Such modifications negatively impact Kelch-like ECH associated protein 1 (KEAP1)-nuclear factor erythroid-derived 2-like (NRF2) signaling axis, wherein succinylation of KEAP1 leads to stabilization of the transcriptional factor NRF2 that drives antioxidant transcriptional responses (Adam et al., 2011, 2014). The antioxidant peptide glutathione is yet another target for succinylation—the modification depletes cellular GSH/GSSG pools, which also results in activation of NRF2-mediated antioxidant responses (Sullivan et al., 2013). Although seemingly beneficial, sustained NRF2 activation may be detrimental to the cell and has been linked to tumorigenesis (Sullivan et al., 2013).

## Signaling through mitochondria-derived free radicals

Mitochondria-produced free radicals and their impact on biological molecules have been extensively studied since the 1950's. For decades, reactive oxygen species (ROS) and reactive nitrogen species (RNS) have been recognized as key contributors to cellular oxidative stress and are detrimental factors in many pathologies and aging (Harman, 1972; Balaban et al., 2005; Wallace, 2005). However, more recent studies established that physiological amounts of free radicals are required to mediate a number of normal cellular processes including several signaling pathways such as hypoxic signaling (Finkel, 2011; Collins et al., 2012; Sena and Chandel, 2012; Chandel, 2014; Shadel and Horvath, 2015). Because a number of outstanding reviews are available on this topic, in this section we will only highlight several key aspects of ROS and RNS-mediated mitochondrial signaling.

### Reactive oxygen species (ROS)

It is commonly accepted that the majority of cellular ROS originates from mitochondrial energy metabolism (Murphy, 2009; Figueira et al., 2013). The flow of electrons through ETC complexes is inevitably linked to a “leakage” of these reducing equivalents, exposing them to molecular oxygen. Partial reduction of O_2_ yields the superoxide anion (${\text{O}}_{2}^{\cdot -}$); it is estimated that ~0.2–2% of O_2_ consumed by mitochondria is converted to superoxide (Figueira et al., 2013). Additional factors, such as full reduction of electron carriers (e.g., under the condition of low ATP production) or elevated NAD^+^/NADH in the matrix (e.g., due to OXPHOS damage), can further stimulate superoxide production.

**Figure 2:**
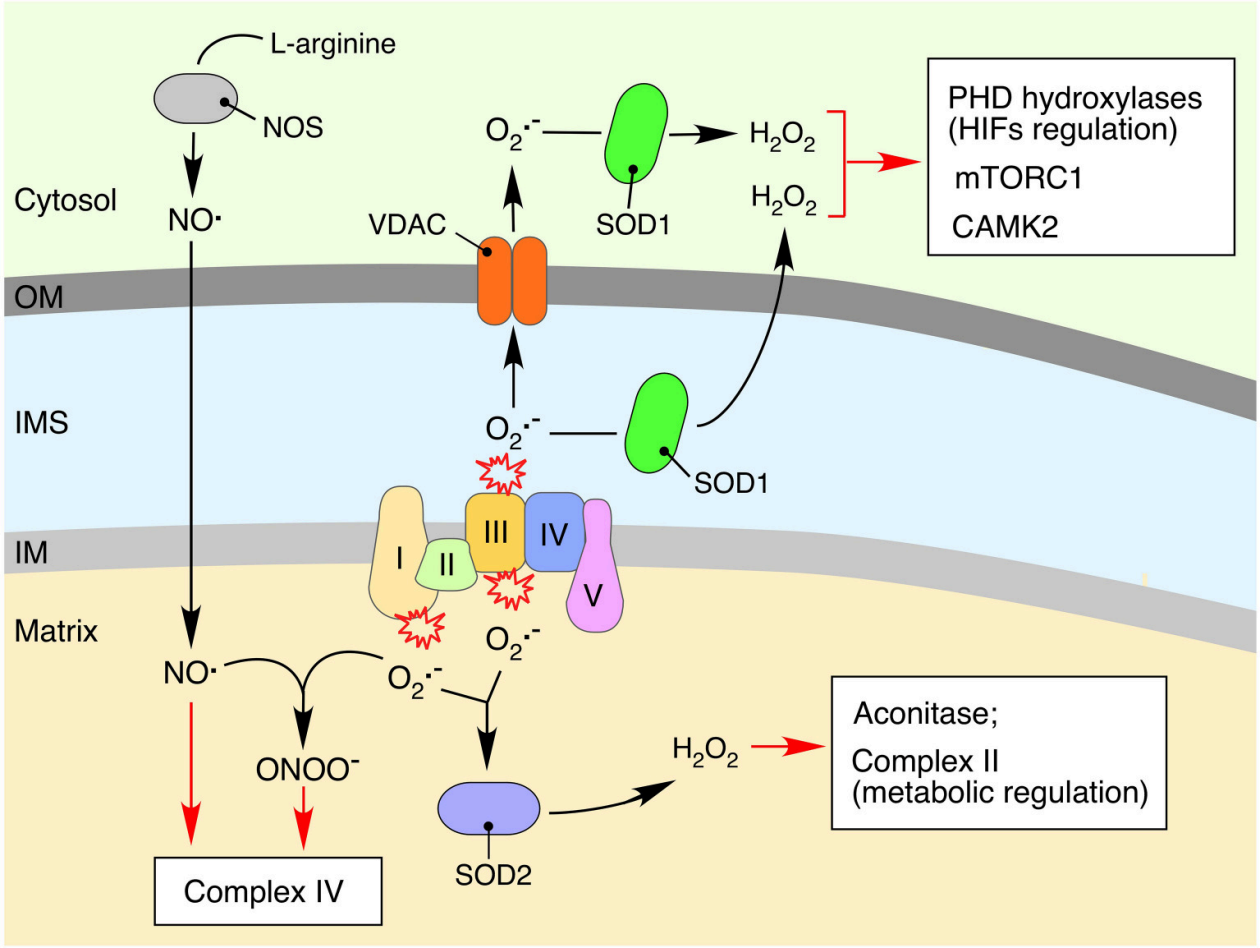
**Impact of mitochondria-derived free radicals on cellular signaling processes**. Reduction of molecular oxygen to the superoxide anion (${\text{O}}_{2}^{\cdot -}$) is an unavoidable consequence of electron leakage associated with the bioenergetic function of respiratory complexes (Figueira et al., 2013). The ${\text{O}}_{2}^{\cdot -}$ produced is quickly converted to hydrogen peroxide (H_2_O_2_) by the mitochondrial matrix-associated manganese (SOD2) or inner membrane space (IMS)-localized copper-zinc (SOD1) superoxide dismutases (Murphy, 2009; Figueira et al., 2013). Accumulation of H_2_O_2_ can affect functioning of mitochondrial proteins, in particular those containing iron-sulfur clusters. Aconitase, a key component of the TCA cycle, is one of the mitochondrial proteins most susceptible to oxidative damage due to the surface exposed iron-sulfur cluster-containing active sites (Armstrong et al., 2004). Mitochondria-derived ${\text{O}}_{2}^{\cdot -}$ and H_2_O_2_ can also impact cytosolic signaling cascades via oxidation of various redox-sensitive proteins including calcium/calmodulin-dependent protein kinase 2 (CaMK2) (Erickson et al., 2008), the mammalian target of rapamycin complex 1 protein kinase (mTORC1), and PHD hydroxylases (Sena and Chandel, 2012). The cytosol-derived nitric oxide radical (NO^.^), a product of arginine conversion by nitric oxide synthases (NOSs), can permeate mitochondrial membranes and inhibit electron transport chain functioning through competitive inhibition of respiratory complex IV or via condensation with ${\text{O}}_{2}^{\cdot -}$ and formation of a potent oxidant, peroxynitrate (ONOO^−^) (Nisoli et al., 2003; Antunes et al., 2004; Figueira et al., 2013).

The two major sites for electron leakage are OXPHOS complexes I and III (Figure 2). Electrons in Complex I leak from the flavin mononucleotide site of the enzyme, thus producing ${\text{O}}_{2}^{\cdot -}$ on the matrix side of the mitochondrial inner membrane; in the case of Complex III, superoxide is produced via the reactive semiquinone intermediate on both matrix and intermembrane space (IMS)-exposed sides of the enzyme (Murphy, 2009; Figueira et al., 2013). The superoxide anion is a short-lived free radical that is readily converted to hydrogen peroxide (H_2_O_2_) by matrix-localized manganese (Mn-SOD, SOD2) or copper-zinc (Cu-Zn SOD, SOD1) superoxide dismutase in the IMS (Figure 2). Nonetheless, elevated levels of ${\text{O}}_{2}^{\cdot -}$ may impair the activity of Fe-S cluster-containing metabolic enzymes, particularly aconitase, whose active site contains a surface-exposed Fe-S cluster that is extremely vulnerable to oxidation (Armstrong et al., 2004). Aconitase inactivation can lead to a malfunctioning TCA cycle and accumulation of intermediary metabolites such as citrate, which will in turn impinge on cellular signaling as described in the previous section.

Unlike superoxide, H_2_O_2_ is a more stable molecule, which can diffuse across mitochondrial membranes. It is thought to be the primary ROS-related signal produced by the mitochondria (Collins et al., 2012). Changes in cellular H_2_O_2_ may affect several signaling-related processes. For instance, reversible oxidation of specific redox-sensitive methionine residues can modulate the activity of calcium/calmodulin-dependent protein kinase 2 (CaMK2), which controls global responses in excitable cells such as cardiomyocytes (Erickson et al., 2008). Similarly, recent data from the yeast genetic model indicate that elevated levels of endogenous mitochondria-borne H_2_O_2_ can modulate the activity of the target of rapamycin (TOR) complex 1 protein kinase (TORC1) (Pan et al., 2011; Bohovych et al., 2016). These data corroborate the results of the earlier *in vitro* analyses, which suggested direct redox-modulation of TORC1 (Sabrassov and Sabatini, 2005). Moreover, H_2_O_2_-triggered alterations in the redox state of metal cofactor containing enzymes can also initiate signaling responses. A well-established example of such a mechanism is the modulation of hypoxic signaling by mitochondria-derived H_2_O_2_ (Guzy et al., 2005; Hamanaka and Chandel, 2010; Sena and Chandel, 2012).

The stability of H_2_O_2_, its ability to traverse cellular membranes, and its propensity to oxidize thiol groups of surface-exposed, low-pKa cysteine residues under physiological conditions makes hydrogen peroxide-mediated post-translational modifications an important molecular event underpinning a wide range of signaling processes in the cell. The selective reactivity of H_2_O_2_ with key cysteine residues of redox-active GTPases, transcription factors, proteases, receptors, kinases, phosphatases, and protein disulfide isomerases can alter the target protein's activity, conformation, or subcellular localization, thereby modulating cellular signal cascades and outputs (reviewed in García-Santamarina et al., 2014; Russell and Cotter, 2015). The reversibility of thiol oxidation (sulfenylation) reactions is assured through the function of electron donor proteins such as thioredoxins, glutaredoxins, and sulfiredoxins, which can all efficiently reduce oxidized cysteines, thus reversing the modulatory effect of thiol oxidation (Brandes and Jakob, 2009). For instance, protein tyrosine phosphatases (PTPs) are among the best-studied components of H_2_O_2_-regulated signal transduction in mammalian cells. Initial evidence of sulfenic acid intermediate formation in response to low micromolar concentrations of H_2_O_2_ in three different PTPs (PTP1, leukocyte antigen-related phosphatase LAR, and vaccinia H1-related phosphatase VHR) was originally reported by Denu and Tanner (1998). Inactivation of the PTPs caused by sulfenylation of the catalytic cysteine can then be followed by secondary modifications like formation of disulfide bonds or sulfenyl amide linkages for further protection against overoxidation (Tanner et al., 2011). An interesting example of redox regulation involving non-catalytic cysteines was described of the human lymphoid tyrosine phosphatase (LYP). Upon oxidizing conditions, a disulfide bond is formed between the catalytic cysteine residue (Cys-227) and the cysteine residues outside of the signature motif (Cys-129) (Tsai et al., 2009). To prevent malproductive reactivation in the reducing environment, Cys-227 forms a disulfide bond with yet another non-catalytic Cys-231, suggesting the possibility of an autoregulatory circuit (Tsai et al., 2009). Multiple outstanding reviews are available on sulfenylation-based activity modulation in various protein kinases (Corcoran and Cotter, 2013; Truong and Carroll, 2013; Russell and Cotter, 2015). The examples include, but are not limited to, the signaling cascades of receptor tyrosine kinases (RTKs), the serine-threonine protein kinase, Akt, mitogen-activated kinases (MAP kinases), cytoplasmic Src (c-Src), inhibitory κB kinases (IKK), and the c-AMP-dependent protein kinase. It should be noted, however, that in many of the aforementioned cases it remains to be determined whether these H_2_O_2_-signaling events involve mitochondria-derived ROS. To date, mitochondria-produced H_2_O_2_ has been implicated in Akt/phosphoinositide-3-kinase (PI3K) signaling via sulfenylation-mediated inactivation of the phosphatase PTEN (Connor et al., 2005). Another example includes the recently described activation of two mitochondria-localized kinases, the Src family tyrosine kinase, Lyn, and the spleen tyrosine kinase, Syk, by mitochondrial ROS (Patterson et al., 2015). Activation of these kinases is important for the proper functioning of several signaling cascades including that of the mitogen-activated protein kinases JNK and Akt (Patterson et al., 2015).

### Reactive nitrogen species (RNS)

The nitric oxide radical (NO^.^) is probably the best-characterized type of RNS. Although mitochondrial origin of NO^.^ remains debated, it is clear that this stable free radical is able to cross mitochondrial membranes and exert several modulatory effects pertinent to mitochondrial signaling (Figure 2). Elevated levels of mitochondrial NO^.^ may alter the mitochondrial bioenergetics status via inhibition of OXPHOS by competing with molecular oxygen for respiratory Complex IV, thereby affecting the cellular ATP/ADP ratio or driving excessive production of superoxide by the ETC (Nisoli et al., 2003; Antunes et al., 2004; Figueira et al., 2013). NO may also drive reversible modification (S-nitrosylation) of specific protein thiols to S-nitrosothiols. Such posttranslational modifications are shown to modulate properties of multiple signaling pathways including those of HIFα and NF-κB (Hess et al., 2005; Nakamura et al., 2013).

Finally, NO^.^ can interact with mitochondrial superoxide and form yet another potent RNS, peroxynitrate (ONOO^−^), which also inhibits the ETC (Antunes et al., 2004) and is likely to trigger signaling responses.

## Mitochondrial signaling through proteins and peptides

The vast majority of polypeptides comprising the mitochondrial proteome are produced by cytosolic translation; newly synthesized polypeptides are imported into the organelle in an unfolded state (Neupert and Herrmann, 2007; Chacinska et al., 2009). However, several core hydrophobic subunits of OXPHOS complexes are produced by mitochondrial translation machinery and need to be stoichiometrically paired with imported polypeptides. This complex nature of the mitochondrial proteome creates a challenging protein folding environment; therefore, it is not surprising that synthesis and assembly of mitochondrial proteome components is a well-orchestrated and highly regulated process (Couvillion et al., 2016). In addition, a large number of metabolic enzymes in the matrix are prone to facile aggregation (Bender et al., 2011). Conditions of mitochondrial stress may perturb stoichiometric equilibrium and cause an accumulation of unassembled and/or misfolded proteins in various mitochondrial sub-compartments and reduce mitochondrial import. Such alterations in mitochondrial protein homeostasis (proteostasis) can be communicated via several mechanisms highlighted below. Furthermore, we will discuss how several mitochondrial polypeptides signal the initiation of an apoptotic program when mitochondrial damage exceeds the repair capacities of a cell.

### Mitochondrial unfolded protein response (UPRmt)

**Figure 3:**
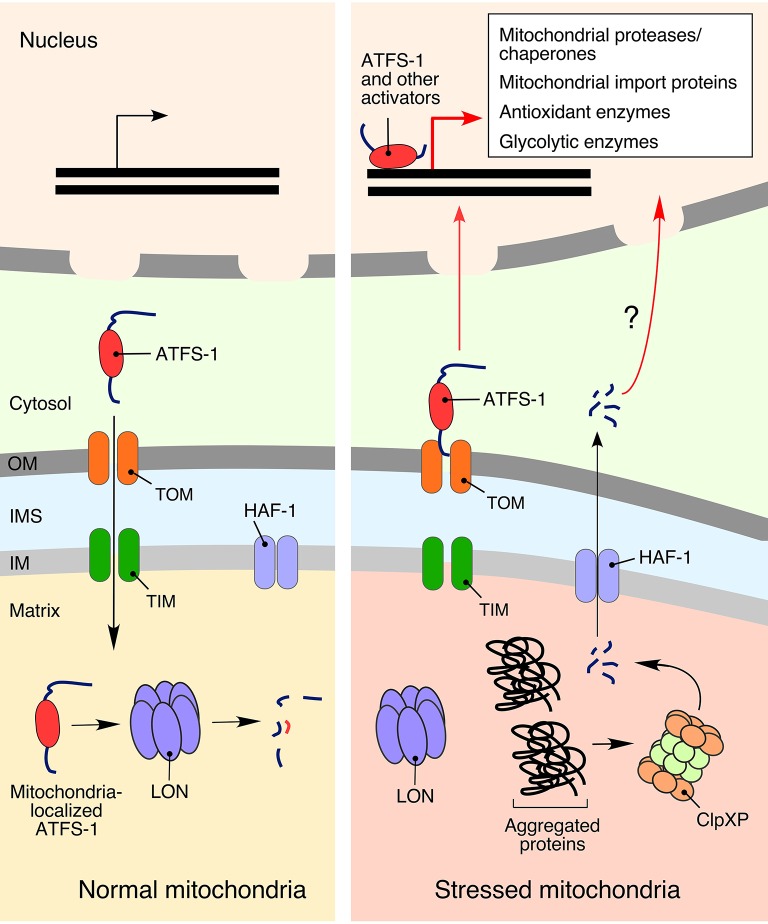
**The mitochondrial unfolded protein response (UPRmt)**. Activation of UPRmt in response to mitochondrial damage is best characterized in the roundworm *Caenorhabditis elegans*, where it is regulated by the activating transcription factor associated with stress 1 (ATFS-1). In the absence of stress stimuli, ATFS1 is transported to the mitochondria and degraded by LON protease (Nargund et al., 2012). Conditions of mitochondrial stress stall mitochondrial import of ATFS-1, allowing a fraction of the protein to translocate into the nucleus and promote expression of more than 400 mitochondrial homeostasis-related genes (Lin and Haynes, 2016). Another, not-so-well defined, activation of UPRmt in *C. elegans* is associated with peptide exporter HAF-1, which translocates signaling peptides generated by the matrix peptidase ClpXP (Haynes et al., 2010).

One of the best-understood responses to mitochondrial proteostatic stress is the mitochondrial unfolded protein response (UPRmt). Activation of UPRmt in higher eukaryotes induces the transcription of genes involved in protein folding and quality control (Pellegrino et al., 2013; Bohovych et al., 2015; Topf et al., 2016), metabolism (Lin and Haynes, 2016), and mtDNA maintenance (Lin et al., 2016). Reciprocally, UPRmt induction is linked to an inhibition of the genes related to both the TCA cycle and OXPHOS components (Nargund et al., 2015). Two key molecular facets of UPRmt were gleaned from studies in the roundworm *Caenorhabditis elegans* genetic model (Figure 3). One aspect involves generation of signaling peptides produced through proteolytic processing of unassembled or misfolded polypeptides by the matrix peptidase ClpXP (Haynes et al., 2010). The peptides are subsequently extruded from mitochondria into the cytosol via the HAF-1 peptide exporter and subsequently activate UPRmt by an unknown mechanism (Haynes et al., 2010, 2013). Another, likely more prominent, component of UPRmt in nematodes is represented by the versatile bZip activating transcription factor associated with stress 1 (ATFS-1) equipped with both a mitochondrial targeting sequence and a nuclear localization signal (Haynes et al., 2010; Nargund et al., 2012). Under normal conditions, ATFS-1 is targeted to mitochondria and inactivated through degradation by the LON protease. However, under conditions of mitochondrial stress that attenuate protein import into the organelle, a portion of AFTS-1 relocates to the nucleus. It was recently reported that UPRmt-associated chromatin remodeling allows nuclear ATFS-1 to bind targeted sequences (Tian et al., 2016) and promote expression of more than 400 genes encoding for proteins involved in mitochondrial proteostasis, metabolism, and innate immunity (Lin and Haynes, 2016).

UPRmt activation in mammalian cells is less understood. It appears to be more complex and stochastic and is achieved via a complex signaling cascade requiring sequential activation of the c-Jun N-terminal kinase, a component of AP-1 transcription factor c-Jun, and finally transcription factors C/EBPβ and C/EBP homologous protein (CHOP) (Pellegrino et al., 2013; Topf et al., 2016), which enhance the expression of so-called canonical UPRmt genes, including those that encode mitochondrial chaperones and proteases (Aldridge et al., 2007; Horibe and Hoogenraad, 2007). Interestingly, a recent study by Seiferling et al. (2016) demonstrated that CLPP appears to play no role in mammalian UPRmt activation.

The other, likely related to the UPRmt, responses reported in mammals include HTRA2 protease-mediated response in the brain (Moisoi et al., 2009) and the StAR accumulation response in steroidogenic cells (Bahat et al., 2014, 2015). The former is a brain-specific transcriptional response caused by the loss of HTRA2 in neuronal cells and a subsequent respiratory defect and accumulation of unfolded proteins in the mitochondria of these cells (Moisoi et al., 2009). The StAR overload response is triggered by elevated levels of the steroidogneic acute regulatory protein (StAR) in the mitochondrial matrix and promotes expression of several mitochondrial proteases to counter the accumulation of this protein within the mitochondria (Bahat et al., 2014, 2015). The molecular aspects of these responses are yet to be clarified.

### Unfolded protein response activated by protein mistargeting (UPRam)

Abnormal accumulation of mitochondrial precursor polypeptides was recently linked to activation of stress responsive pathways in the cytosol (Topf et al., 2016). Two recent publications reported a novel type of mitochondrial proteostatic response termed mPOS (*m*itochondrial *p*recursor *o*ver-accumulation *s*tress) (Wang and Chen, 2015; Wrobel et al., 2015). The mPOS is associated with defects in mitochondrial import machinery, and inner membrane integrity and function. The response appears to modulate two distinct cellular activities: (1) cytosolic protein synthesis; and (2) an unfolded protein response activated by protein mistargeting (so-called UPRam). The latter significantly differs from the canonical mitochondrial unfolded protein response (UPRmt), a pathway triggered by impaired mitochondrial proteostasis (Wrobel et al., 2015). Protein synthesis regulation by mPOS involves a global increase in cap-independent translation (and reciprocal reduction of cap-dependent translation), as well as modulation of ribosomal biogenesis (Wang and Chen, 2015; Wrobel et al., 2015). The signaling mechanisms underpinning these events remain to be determined.

A similar modulation of protein biogenesis is also related to antibiotic-induced defects in mitochondrial translation. Stalling of mitochondrial ribosomes in mouse embryonic fibroblasts by treatment with the mitochondrial translation inhibitor, actinonin, activates cellular proliferation signaling pathways such as that of p53 and MAP kinase (Richter et al., 2013).

### PINK1-Parkin signaling relay

**Figure 4:**
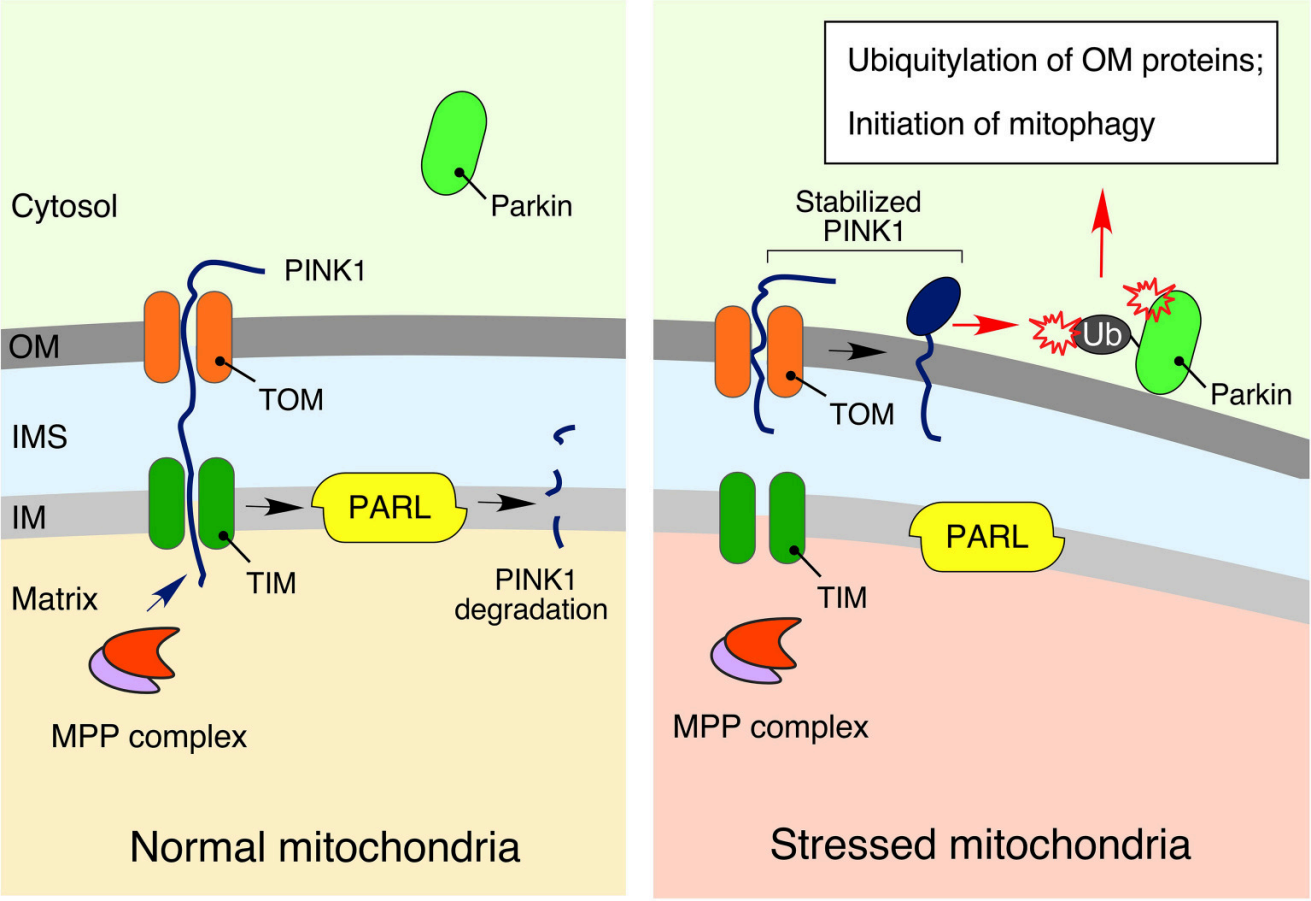
**The PINK1-Parkin signaling relay**. Under physiological conditions, phosphatase and tensin homolog (PTEN)-induced ubiquitin kinase 1 (PINK1), a key regulator of mitophagy and mitochondrial turnover, is imported into the mitochondria via the translocases of outer (TOM) and inner (TIM) membranes. It is subsequently degraded by presenilin associated rhomboid-like protease (PARL) in the IM (Jin et al., 2010). However, in response to mitochondrial damage, stabilized PINK1 accumulates on the outer membrane (OM), promoting recruitment, and activation of the E3 ubiquitin ligase parkin, followed by ubiquitylation of OM-associated proteins, and initiation of mitophagy (Kondapalli et al., 2012; Kane et al., 2014; Kazlauskaite et al., 2014; Koyano et al., 2014; Ordureau et al., 2014). More details are available in the text.

Higher eukaryotes possess yet another elegant mechanism to signal mitochondrial distress and promote removal of dysfunctional mitochondria via selective autophagy (mitophagy). This mechanism, known as the PINK1-Parkin relay (Figure 4), composed of the phosphatase and tensin homolog (PTEN)-induced ubiquitin kinase 1 (PINK1) and the E3 ubiquitin ligase Parkin, mediates poly-ubiquitylation of damaged mitochondria thereby priming mitophagy (Sarraf et al., 2013; Lazarou et al., 2015). Similar to ATFS-1, PINK1 is imported into mitochondria under normal conditions, wherein the protein is promptly degraded by the IM resident presenilin associated rhomboid-like protease (PARL) (Jin et al., 2010). Conditions of mitochondrial stress that perturb protein import into the organelle cause the stabilization and accumulation of PINK1 on the mitochondrial outer membrane (OM), where this kinase drives phosphorylation of Parkin as well as ubiquitin (Kondapalli et al., 2012; Kane et al., 2014; Kazlauskaite et al., 2014; Koyano et al., 2014; Ordureau et al., 2014). These actions promote: (1) mitochondrial retention of Parkin (Ordureau et al., 2014); (2) subsequent assembly of poly-ubiquitin chains on various OM proteins (Sarraf et al., 2013); and (3) activation of autophagosome-stimulating protein TANK-binding kinase 1, TBK1 (Heo et al., 2015). Of note, genetic mutations in the components of this pathway have been linked to familial cases of neurodegenerative diseases such as Parkinson's disease (Pickrell and Youle, 2015) and amyotrophic lateral sclerosis (Maruyama et al., 2010; Wong and Holzbaur, 2014; Cirulli et al., 2015).

Interestingly, the recently described mitochondria-derived vesicles (MDV) quality control pathway that appears to mediate crosstalk between mitochondria and either peroxisomes or lysosomes (Neuspiel et al., 2008; Sugiura et al., 2012) also seems to rely on the mitophagy-independent function of the PINK1-Parkin relay (Sugiura et al., 2012).

### Cytochrome *c*

Mitochondria are intimately involved in the control of apoptotic signaling cascades. In particular, initiation of apoptosis is associated with release of the IM and IMS proteins such as cytochrome *c*, apoptosis inducing factor AIF, second mitochondria-derived activator of caspase protein (SMAC), serine protease high temperature requirement protein A2 (HTRA2), and endonuclease G. Upon their release from irreversibly damaged mitochondria through the β-cell CLL/lymphoma 2 (Bcl2) family proteins-mediated mitochondrial outer membrane permeabilization, these proteins serve as signaling molecules to regulate and propel cell death processes (Tait and Green, 2010).

The small nuclear-encoded electron carrier cytochrome *c* plays one of the key roles in programmed cell death—its release is a critical event necessary to initiate an apoptotic program (Liu et al., 1996; Hüttemann et al., 2011). This rapid feed-forward process commences with disassociation of cytochrome *c* from its complexes with the mitochondria-specific phospholipid cardiolipin following the protein's detachment from the IM (Goldstein et al., 2000; Ott et al., 2002; Garrido et al., 2006). While cytochrome *c*-independent forms of cell death have been reported (e.g., see the review by Sevrioukova, 2011), here we will focus on mitochondrial signaling via this molecule.

Accumulation of released cytochrome *c* in the cytosol triggers the formation of the apoptosome—a wheel-like structure containing seven molecules of apoptotic protease-activating factor 1 (APAF-1) equaled by cytochrome *c*. The apoptosome complex activates the key apoptotic factor, caspase 9, which subsequently promotes the execution of the apoptotic program (Reubold and Eschenburg, 2012). Of note, cytochrome *c*-induced caspase activation is not always associated with cell death. Examples of the vital processes requiring cytochrome *c* release include platelet formation, β-cell proliferation, and monocyte-macrophage differentiation (Garrido et al., 2006). Similarly, in *Drosophila melanogaster*, cytochrome *c* serves as a crucial signaling molecule in caspase-mediated sperm cell development (Nagata, 2016).

### Mitochondria-derived peptides

Just like in bacteria, the initiation of mitochondrial translation requires N-formylmethionine-tRNA (Ott et al., 2016). Mitochondria-derived N-formylated peptides have long been known as potent chemoattractants for neutrophils (Carp, 1982). Further studies demonstrated binding of the polymorphonuclear neutrophils (PMN)-expressed formyl peptide receptors (FPRs) by native and synthetic peptides originated from mitochondrial Complex I subunits 4 and 6, Complex IV subunit 1 (Rabiet et al., 2005), and cytochromes *b* (Mukai et al., 2009; Seki et al., 2011) and *c* (Hokari et al., 2012). Subsequent activation of FPRs involves multiple signaling cascades controlling calcium flux, p38 kinase activation, and cytokine production (Rabiet et al., 2007). It is noteworthy that the release of formylated mitochondrial peptides following trauma is associated with inflammation, thus potentially linking injury to non-infective systemic inflammatory response syndrome and sepsis (Zhang et al., 2010).

The mammalian mitochondrial genome is known to encode only 11 proteins (Ott et al., 2016). However, this number might be extended due to the recent discovery of short open reading frames (sORFs), which encode so-called mitochondrial-derived peptides (MDPs) (Lee et al., 2016). Perhaps the best-characterized MDP is a 24-amino acid long peptide named humanin—which was originally shown to bind to the pro-apoptotic factor Bax in a manner similar to that of its nuclear-borne homolog; therefore suggesting a potential regulatory role in cell death (Guo et al., 2003). More detailed reviews of various humanin-associated cytoprotective effects can be found elsewhere (Lee et al., 2013).

Another MDP called MOTS-c (mitochondrial open reading frame of the 12S rRNA-c) was discovered more recently. The activity of this 16-amino acid long peptide has been linked to the regulation of muscle energy expenditure and insulin sensitivity. MOTS-c activates a metabolic/signaling axis comprised of the one-carbon folate cycle and AMPK, thereby providing an obesity-protective effect (Lee et al., 2015). Interestingly, the most recent study by Cobb et al. (2016) reported identification of six sORFs originating from the same genetic locus as humanin which have been named small humanin-like peptides (SHLPs). The signaling roles of SHLPs remain to be elucidated.

## Other mitochondria-derived signals

In this section, we will survey several additional molecules that cannot be placed into any of the above groups of mitochondria-borne signals. This does not imply that the following molecules are not linked to the pathways described above. Likewise, these signals do not appear to function on their own and are well intertwined with the signals described above and with each other. For instance, signaling via changes in mitochondrial and intracellular calcium levels is linked to ROS-mediated communication, as well as signals arising from defects in the mitochondrial genome.

### Calcium ions

The mitochondria can be viewed as an efficient Ca^2+^ sink that assists the endoplasmic reticulum (ER) and lysosomes in control of cellular metabolism and death via apoptosis, necrosis, and autophagy (Rizzuto et al., 2012). Active flux of Ca^2+^ between the ER and mitochondria is driven via the mammalian mitochondria-associated membranes (MAMs) or yeast's ER-mitochondria encounter structures (ERMES). A decrease of Ca^2+^ levels in the mitochondria leads to a rapid drop in ATP production, followed by activation of the AMPK signaling pathway, resulting in subsequent initiation of autophagy (La Rovere et al., 2016). Conversely, mitochondrial Ca^2+^ overload promotes opening of the mitochondrial permeability transition pore (mPTP), a non-specific transporting channel in the IM resulting in mitochondrial membrane depolarization, swelling, OM rupture, and release of IM and IMS residing pro-apoptotic factors (Bhosale et al., 2015; Takeuchi et al., 2015). Increased levels of Ca^2+^ in the cytosol can activate various soluble Ca^2+^-binding proteins such as calmodulin, which in turn can bind to various targets including calmodulin-dependent CaMK protein kinases, myosin light chain kinase MLCK, and calcinuerin—the key regulators of a plethora of cellular processes (Tidow and Nisse, 2013). Of note, excessive accumulation of Ca^2+^ and failure to clear cytoplasmic Ca^2+^ have been linked to necrosis initiation (Rizzuto et al., 2012).

### Mitochondrial DNA and replication defects

The first insights into the impact of mitochondrial DNA (mtDNA) depletion or lesions on nuclear gene expression in mammalian cells were reported by Avadhani's lab in the late 1990's and linked these defects to decreased Ca^2+^ buffering capacity of the mitochondria and ultimately cytosolic calcium accumulation (Biswas et al., 1999; Amuthan et al., 2001). As outlined in the previous section, elevated levels of cytosolic ${\text{Ca}}_{2}^{+}$ trigger the activation of calcineurin and downstream transcriptional factors such as NF-κB, CHOP, and extracellular signal-regulated kinase 1 (ERK1) (Butow and Avadhani, 2004).

mtDNA, with its circular loop and numerous regions of non-methylated DNA (CpG islands), partially resembles bacterial genomes (Fang et al., 2016). Similar to bacterial pathogen-associated molecular patterns, mtDNA along with formylated peptides (see above) has been described as mitochondria-derived damage-associated molecular patterns (DAMPs) that are capable of PMNs stimulation via Toll-like receptor 9 (TLR9) and, subsequently, the p38 MAP kinase signaling cascade (Zhang et al., 2010). In addition, accumulation of mtDNA in the cytosol can activate the DNA-specific sensor, cGAS, which promotes signal transduction via the STING-TBK1-interferon regulatory factor 3 (IRF3)-dependent pathway, thereby increasing interferon-stimulated gene expression. This chain of events ultimately results in activation of an antiviral immune response (West et al., 2015). Under apoptotic conditions, oxidized mtDNA also appear to activate nucleotide-binding domain leucine-rich repeat family, pyrin domain containing 3 (NLRP3) inflammasome, and IL-1β production (Shimada et al., 2012). It is noteworthy that the NLRP3 inflammasome itself is associated with mitochondria through its interactions with mitochondrial antiviral signaling protein MAVS, mitochondrial OM GTPases mitofusins, caspase-like apoptosis regulatory protein c-FLIP, and the phospholipid cardiolipin (Elliot and Sutterwala, 2015).

mtDNA maintenance and replication requires a complex network of factors encoded by both the nuclear and the organelle's genomes (Ott et al., 2016). Mutations leading to the depletion of mtDNA have a profound effect not only on mitochondrial health, but also on the overall course of various diseases in human patients (Young and Copeland, 2016). For instance, mutated forms of the mtDNA helicase, TWINKLE, are associated with mitochondrial myopathy (MM) and infantile onset spinocerebellar ataxia (IOSCA). Mouse models for MM and IOSCA display a significant number of mtDNA replication defects upon modulation of purine and serine/glutathione biosynthesis (Nikkanen et al., 2016). Previous studies in the late-onset MM mouse model also demonstrated a fasting-like transcriptional response via the AKT1-PI3K signaling cascade upon disease progression. Particularly, increased production of the starvation-associated fibroblast growth factor 21 (Fgf21) was detected in the mutation-carrying animals, which, in turn, caused profound changes to lipid metabolism, including resistance to high-fat diet (Tyynismaa et al., 2010). CHOP10 is another transcription factor activated upon defective mtDNA expression. However, this response does not appear to be associated with the UPRmt and is likely a manifestation of a general stress response (Michel et al., 2015).

### Cardiolipin

Cardiolipin (CL) is a unique non-bilayer phospholipid specific to mitochondrial membranes. Mounting evidence indicates that CL contributes to a plethora of mitochondrial processes including signaling events required to initiate mitophagy and apoptotic cell death (Lu and Claypool, 2015). Under normal physiological conditions, CL is almost exclusively localized to the IM. However, mitochondrial stress stimuli trigger random CL translocation to the OM, and this event serves as a signal to activate the autophagy machinery via CL binding to its central component, microtubule-associated protein 1 light chain 3 (LC3) (Chu et al., 2013; Kagan et al., 2015). Mitophagy-specific induction by CL requires the phospholipid's remodeling by tafazzin, an evolutionary conserved monolyso-CL transacylase (Hsu et al., 2015). Mutations in tafazzin are linked to the human multisystemic disorder, Barth syndrome (Lu and Claypool, 2015). Studies in lymphoblastoid cells from Barth syndrome patients reveal an important role for CL in the translocation of the pro-apoptotic protease, caspase 8 (Gonzalvez et al., 2008).

Release of mitochondria-associated pro-apoptotic factors is believed to require selective CL oxidation by cytochrome *c* (Kagan et al., 2005; Choi et al., 2007); *in vitro* experiments suggest that cytochrome *c* might acquire its peroxidation activity upon interaction with the phospholipid (Basova et al., 2007). The CL-mediated decision (mitophagy vs. apoptosis) likely depends on the extent of CL externalization (Lu and Claypool, 2015).

Finally, CL is implicated as a potent immunogenic factor. First, it is capable of activating the NLRP3 inflammasome via direct binding to the NLRP3 component. As such, this event leads to caspase 1 activation and further production of the proinflammatory cytokines, IL-1β, and IL-18 (Iyer et al., 2013). Another exciting aspect is the ability of liposomes and mitochondria with CL-containing surfaces to be efficiently recognized and engulfed by macrophages in a CD36-dependent manner *in vitro* (Balasubramanian et al., 2015). It will be interesting to ascertain if a similar process occurs *in vivo*.

## Concluding remarks

In this review, we have surveyed several mitochondrial signals that regulate the organelle's biogenesis and stress-response pathways. One cannot help but notice that a number of these signals and responses appear to be triggered by similar, if not the same, homeostatic insults. How mitochondria-borne signals are interrelated, and if there is any signaling hierarchy, remains to be clarified. It will be important to gain further mechanistic understanding into how mitochondrial stress signals are propagated and transferred. Because a number of the insights described above were gleaned from studies in model organisms or *in vitro* cell cultures, it will be equally important to determine the extent and magnitude of such signaling responses in organs and tissues. These findings may help to better understand a tissue-specific nature of certain mitochondria-related pathologies.

Another exciting question is whether these signals can be manipulated to increase mitochondrial stress tolerance and achieve therapeutic benefits in mitigating disease-related states or extending lifespan. In that respect, recent findings such as NAD^+^ repletion-mediated improvement of mitochondrial function and lifespan in mice (Zhang et al., 2016) or potential therapeutic effects of hypoxic preconditioning in the rodent model of mitochondrial disease (Jain et al., 2016), as well as ones of mitochondria-targeted antioxidants in the murine model of Huntington's disease (Xun et al., 2012) are encouraging and create a solid premise for future translational studies. Likewise, targeting mitochondrial signals or signaling pathways emerges as a prospective theranostic paradigm in treatment of various cancers (Zong et al., 2016).

## Author contributions

IB analyzed the literature and co-wrote the manuscript. OK conceived the idea, analyzed the literature, co-wrote the manuscript, and designed the figures.

### Conflict of interest statement

The authors declare that the research was conducted in the absence of any commercial or financial relationships that could be construed as a potential conflict of interest.
